# Behavioral and sociodemographic predictors of anxiety and depression in patients under epidemiological surveillance for COVID-19 in Ecuador

**DOI:** 10.1371/journal.pone.0240008

**Published:** 2020-09-30

**Authors:** Clara Paz, Guido Mascialino, Lila Adana-Díaz, Alberto Rodríguez-Lorenzana, Katherine Simbaña-Rivera, Lenin Gómez-Barreno, Maritza Troya, María Ignacia Paez, Javier Cárdenas, Rebekka M. Gerstner, Esteban Ortiz-Prado

**Affiliations:** 1 School of Psychology, Universidad de Las Américas, Quito, Ecuador; 2 Faculty of Health Science, One Health Research Group, Universidad de Las Américas, Quito, Ecuador; 3 National Department of Mental Health, Ministry of Public Health in Ecuador, Quito, Ecuador; 4 Department of Sicoethics, Pontificia Universidad Católica del Ecuador, PUCE, Sede Santo Domingo, Quito, Ecuador; University of New South Wales, AUSTRALIA

## Abstract

Ecuador has been one of the most affected countries by the Corona Virus Disease 19 (COVID-19) pandemic, by April 2020 this country presented the highest rates of mortality in Latin America. The purpose of the present study was to identify behaviors during confinement and sociodemographic variables associated with the mental health status of confirmed or suspected COVID-19 patients who were part of the epidemiological surveillance program in Ecuador that included mandatory confinement and self-isolation. A cross-sectional study was performed from March 22^th^ to April 18^th^, 2020 using an online survey. The survey collected socio-demographic information and severity of depressive symptoms using the Patient Health Questionnaire-9 and anxiety symptoms through the Generalized Anxiety Disorder-7. A total of 759 patients completed the questionnaire, 20.3% presented moderate to severe symptoms of depression and 22.5% moderate to severe symptoms of anxiety. Being a woman and from the Coastal region were risk factors. Exercising, maintaining daily routines, and keeping informed about the COVID-19 but limiting to an hour was associated with better mental health. Regression analysis indicated that the mentioned behaviors explained approximately 17% of the variance for depression sum scores and 11.8% of the variance for anxiety sum scores while controlling for gender and region. Understanding the association between sociodemographic variables and psychological states in patients with COVID-19 is relevant to tackle future public mental health problems and to implement health policies that are intended to palliate further psychiatric complications. Promotion of modifiable behaviors such as exercising, maintaining daily routines, and keeping informed about the COVID-19 but limiting to less than an hour is recommended.

## Introduction

The first cases of Corona Virus Disease 2019 (COVID-19) emerged in Wuhan China on late December 2019 [1]. One month later, the presence of this disease was declared a public health emergency of international concern by the general director of the World Health Organization (WHO). As of April 30, 2020, the WHO [2] registered a total of 3,090,445 COVID-19 confirmed cases worldwide and 217,769 confirmed deaths. In total, 213 countries, areas, or territories were affected by the COVID-19 by then. The rapid spread of the virus throughout the world has forced local and national administrations to take unprecedented measures to reduce the impact of this pandemic [3]. The measures included active surveillance for suspected cases, self-isolation or social distancing, restrictions for travel and transportation, and even shutting down country boarders [4].

In this scenario, isolation, economic recession, pressure on health professionals and inequities in access to care and disparities in outcomes according to socioeconomic status have resulted in reactive anxiety, depression, and even the presence of suicide attempts in the general population [5, 6]. The most vulnerable groups to develop these symptoms seems to be women, young adults (> 35 years), people with low income or unemployed and singles [7]. This situation has led to actions by multiple nations aimed at understanding the mental health consequences for the general population as well as the most vulnerable groups under the pandemic conditions as a priority for researchers in the area [8].

Two studies conducted in China [9, 10] using a survey format directed towards a general adult population in February 2020, indicated that 28–35% of the participants had experienced symptoms of generalized anxiety disorders and 16–20% symptoms of depression. In the same country, the study conducted by Wang, Di, Ye, & Wei [11] indicated that gender, age, education and type of job are some demographic related factors that are associated with depressive and anxiety symptoms. Females presented higher levels of anxiety as well as people above 40 years old. Additionally, depression levels were higher in people with a bachelor’s degree when compared with people with a master’s degree, and industrial service workers had a higher probability of having depression in comparison to highly qualified professionals. When considering the psychological effects of the pandemic for those diagnosed with COVID-19, that group presented with a high prevalence of depression (29.2%) and anxiety (20.8%) in a recent study [12].

Not only sociodemographic variables have been studied in relation to the presence of psychological symptoms during the COVID-19 pandemic; some studies have also identified the association of the presence of modifiable behavioral factors on the development and / or maintenance of mental health problems like anxiety and depression [13, 14]. Huang et al. [9] identified that focusing on information about COVID-19 for three or more hours per day may act as a risk factor for the presence of anxiety. In Germany [15], a similar study found that revising this information for more than two hours and a half and/or more than seven times per day is related with critical symptoms of depression and anxiety and increased phobic fear of COVID-19. However, Zhong et al. [16]. reported that having more information about COVID-19 was significantly associated with a lower probability of developing negative attitudes during confinement. Another variable which has been associated with mental health is the engagement in physical activity. The number of days that the person exercise during the confinement has been related with lower levels of depression and anxiety [7], but also the reduction of regular physical activity has been related with greater levels of anxiety concerning with COVID-19 topics [17]. Moreover, some other behaviors have been recommended to reduce psychological distress during the confinement based on previous evidence. Altena et al. [18] have proposed the maintenance of daily routines to better coping with the psychological consequences of the pandemic. Isolation is one of the most challenging situation under these circumstances, and as a result Brooks et al. [6] have indicated that the activation of remote social networking can help to reduce immediate anxiety and long-term distress. All these modifiable behaviors are some of the considerations included as part of the messages that should be promoted in communications to support mental health of the population prepared by the WHO Department of Mental Health and Substance Use [19].

Most of the reported studies about the mental health impact of the COVID-19 have been conducted in China, which is understandable since this was the first country affected by the pandemic. Despite the usefulness of these data, demographic and inter-individual characteristics are determining factors in the psychological responses of different populations to a large-scale stressful event such as COVID-19 [20]. For this reason, it is essential to increase the volume of studies evaluating the impact of this pandemic in each of the affected countries, especially in countries such as Ecuador, which has been one of the most affected countries by the pandemic, by April 2020 it presented the highest rates of mortality in Latin America [21].

We have reported briefly a comparison of depression and anxiety symptoms between persons confirmed and suspect of COVID-19 in Ecuador [22], however we have not deepen on the analysis of the variables related to the presence of these symptoms. The purpose of the present study is to analyze that data in order to assess the mental health symptoms and associated sociodemographic variables and behaviors during the confinement of people who were part of the epidemiological surveillance program for COVID-19 established by the Ecuadorian Ministry of Public Health during March and April 2020.

## Materials and methods

### Study design

A cross-sectional analysis was designed to evaluate the self-reported mental health symptoms in relation to sociodemographic variables and modifiable behaviors presented during the confinement by persons under the epidemiological surveillance program for COVID-19 in Ecuador from March 22^th^ to April 18^th^, 2020.

### Setting

This study was conducted in Ecuador, a country located in South America split in four main regions: 1) the Coastal region, 2) the Highland or Sierra region, 3) the Amazon region and 4) the Insular region. Coastal region presented higher mortality rates than the other regions as consequences of late implementing restrictive measures of social distancing and limited capacity of health services [23]. The population of Ecuador was estimated to be 17,510,643 inhabitants based on the latest available projections for 2020 [24].

### Participants and procedures

This ongoing monitoring by the health department was the result of contact tracing strategy deployed by the Ministry of Public Health (MoPH) among confirmed or suspected COVID-19 patients in Ecuador. The Mental Health Department at the MoPH in Ecuador lead the active surveillance with mental health personnel from the districts, which deployed the online self-reporting tool among patients. A non-probabilistic sampling strategy was used from the beginning of the study until quota was reached. Using the methodology described by Ortiz-Prado et al. 2020 [23], a total number of completed answers reached 856 patients in order to achieve 99% confidence. The included patients in this study were adults (> = 18 years old) who were under epidemiological surveillance for being diagnosed with or suspected to have COVID-19 following exposure, and who voluntarily responded to the survey.

### Measures

#### Sociodemographic information and behaviors during the confinement

The main sociodemographic variables were recorded such as age, gender, employment, place of residence and educational attainment. The behaviors during the confinement were collected through four specific questions 1) are you maintaining regular schedule to eat and to sleep? 2) are you exercising regularly? 3) how many people are you interacting daily through online social networks? 4) how much time are you spending daily to get information about COVID-19? Psychological and emotional status were assessed using the following measures.

#### Patient Health Questionnaire (PHQ-9) [25]

It is a brief self-report questionnaire to measure the severity of depressive symptoms in adults. Scores from one to four indicate presence of minimal symptoms, from five to nine mild, from 10 to 14 moderate, from 15–19 moderately severe, and from 20–27 severe depression. The psychometric properties of the measure are good, notable for test-retest reliability of 0.84 and internal consistency of 0.89 [25]. The Spanish version of this measure, which has comparable psychometric properties (Cronbach α = .89) with the original version, was used in the present study [26]. The optimal cut-off score for diagnosing depression with the Spanish version of this measure has been proposed to be 12 [27].

#### Generalized Anxiety Disorder (GAD-7) [28]

It is a seven-item measure that assess the presence and severity of generalized anxiety disorder in adults. Total sum scores from one to four indicate presence of minimal symptoms, from five to nine mild, from 10 to 14 moderate, and greater than 15 severe anxiety. The measure has presented excellent internal consistency (Cronbach α = .92) and good test-retest reliability (intraclass correlation = .83). In the present study, the Spanish version [29] of the measure was used. This version has presented good psychometric properties (Cronbach α = .93, test-retest correlation = 0.92), and 10 has been identified as an optimal cut-off point to detect generalized anxiety disorder as diagnosis.

### Data analysis

Descriptive statistics were conducted to characterize the sample. Frequencies and percentages were calculated for each sociodemographic variable (gender, age group, education, employment, region and localization) and for each behavior during confinement (maintenance of daily routines, exercising, social contact and time focusing on information about the COVID-19). Mean and standard deviations were calculated for the total scores retrieved from the sum scores of the PHQ-9 and the GAD-7. The scores were coded categorically to express the severity of anxiety and depression, according to the indications presented in the measures section above. Frequencies and percentages were calculated for each severity level. Also, the percentage of participants presenting with both depression and anxiety was calculated. The possible associations between sociodemographic variables and psychological symptoms (total sum scores of the PHQ-9 and GAD-7) were tested through t-test for variables with two levels and analyses of variance (ANOVA) for variables with more than two levels. The same procedure was used to test possible associations between behaviors during the confinement and psychological symptoms. Two-step hierarchical regression analyses were performed to determine the contribution of each variable to GAD-7 and PHQ-9 total sum scores separately. In the first step we included the sociodemographic variables presenting significant differences and in the second step we added the behaviors during the confinement presenting significant differences while controlling for the sociodemographic variables previously identified. All tests were two-tailed, and significance level was set up at *p* < 0.05. All the statistical analysis were conducted using R [30]. The minimal data set to replicate these analyses is included as supporting information (S1 Dataset).

### Ethical consideration

According to the Ecuadorian law and international good clinical and research practices observational studies that do not put in risk patients’ rights are exempted to obtain full ethical approval. This study presents a secondary data analysis of Ortiz-Prado et al. 2020 [23], which received ethical exemption from the Universidad de Las Américas Ethics Committee (CEISH) because it used only anonymized, un-identifiable information of the patients. Follow-up phone calls and the information retrieved was obtained by health professionals who offer standard care treatment for confirmed and suspected patients with COVID-19. The information from those reports were anonymized, ensuring no susceptible information was shared to any member of our research team. All the participants provided informed consent after reading the information for the study and before to start to use the self-reporting tool by clicking in the button “Accept”.

## Results

In total, 759 persons in epidemiological surveillance completed the survey, 51.9% were females. The mean age was 37 years (*SD* = 11.01) ranging from 18 to 94 years. Respondents were from the three main regions in Ecuador: Coastal, Sierra and Amazon. At the moment of data collection, the number of persons under epidemiological surveillance in the Insular region was minimal. Information in detail about sample characteristics can be found in Table 1.

**Table 1:** Sociodemographic information and group differences of GAD-7 and PHQ-9 sum scores.

Variable		*n* (%)	GAD7			PHQ9		
			Mean (SD)	Test	*p*	Mean (SD)	Test	*p*
				*F/t* ^a^			*F/t* ^a^	
Gender				5.02	< .001		5.38	< .001
	Male	365 (48.1)	5.24 (4.97)			4.79 (5.04)		
	Female	394 (51.9)	7.09 (5.17)			6.83 (5.43)		
Age (years)				1.05	.55		0.13	.87
	(18–35)	413 (54.4)	6.44 (5.16)			5.76 (5.21)		
	(36–53)	278 (36.6)	5.96 (5.17)			5.94 (5.40)		
	(older than 53)	68 (9)	5.71 (5.05)			6.04 (5.92)		
Education				-1.16	0.25		-1.11	0.27
	Less than 12 years of formal education	204 (26.9)	5.82(5.60)			5.47(5.94)		
	12 or more years of formal education	555 (73.1)	6.34(4.98)			5.99(5.10)		
Employment				1.57	.12		1.56	.19
	Employed	582 (76.7)	6.33 (5.22)			5.89 (5.31)		
	Unemployed	87 (11.5)	5.54 (4.87)			5.11 (5.14)		
	Studying	42 (5.5)	7.12 (5.28)			7.21 (5.68)		
	Other	48 (6.3)	4.98 (4.43)			5.46 (5.62)		
Region				4.013	0.01		8.81	< .001
	Coastal	451 (59.4)	6.61 (5.22)			6.49 (5.46)		
	Sierra	237 (31.2)	5.75 (4.96)			5.10 (4.96)		
	Amazon	71 (9.4)	5.10 (5.15)			4.28 (5.18)		
Localization								
	Rural	149 (19.6)	6.02 (5.31)			4.85 (4.82)		
	Urban	610 (80.4)	6.24 (5.12)			6.09 (5.43)		

^a^ *t* statistic for variables with two levels and *F* statistic for variables with more than two levels

### Levels of anxiety and depression

The mean level of anxiety measured by the GAD-7 was 6.2 (*SD* = 5.15); 41.9% (*n* = 318) had none or minimal presence of symptoms of anxiety, 35.6% (*n* = 270) had sum scores indicating mild, 14.6% (*n* = 111) moderate, and 7.9% (*n* = 60) severe anxiety. The mean level of depression measured by the PHQ-9 was 5.85 (*SD* = 5.34); almost half of the participants (47.4%; *n* = 360) had none or minimal presence of symptoms of depression, 32.3% (*n* = 245) had sum scores indicating mild, 12.3% (*n* = 93) moderate, 5.5% (*n* = 42) moderately severe, and 2.5% (*n* = 19) severe depression. The last item of the PHQ-9 is an indicator of suicide ideation; 6.6% (*n* = 50) responded positively to this item, indicating the presence of risk. In total, 9.1% (*n* = 69) of the participants presented both depression and anxiety classified using the cutoff scores proposed for each measure (PHQ-9 = 12, GAD-7 = 10), indicated in the measures section above.

### Sociodemographic variables and psychological symptoms

Mean differences of GAD-7 and PHQ-9 sum scores according several sociodemographic variables can be found in Table 1. The results of the univariate analysis indicated that only gender and region presented statistically significant mean differences for GAD-7 and PHQ-9 sum scores. Males presented lower mean scores of GAD-7 and PHQ-9 than females. For post-hoc analysis of scores according region where patient was living, pairwise comparisons Fisher’s Least Significant Difference (LSD) test were used. The results indicated significant differences of GAD-7 total scores of persons from Coastal and the other regions (Coastal vs. Sierra mean difference = - 0.86, *p* = .04; Coastal vs. Amazon mean difference = -1.51, *p* = .02). No significant mean differences were found of persons from Sierra vs. Amazon region (mean difference = - 0.64, *p* = .35). The same pattern was found for PHQ-9 total scores (Coastal vs. Sierra mean difference = - 1.38, *p* = .001; Coastal vs. Amazon mean difference = - 2.28, *p* = .001; Sierra vs. Amazon mean difference = - 0.81, *p* = .25). Patients from Coastal region presented higher levels of depression and anxiety.

### Behaviors during the confinement and psychological symptoms

Mean differences of GAD-7 and PHQ-9 sum scores according each behavior during the confinement can be found in Table 2. Significant mean differences were found for the levels of the variables: maintenance of daily routines, exercising and time focusing on information about the COVID-19. Those patients that maintained daily routines presented lower sum scores in GAD-7 and PHQ-9 than those who did not maintain a regular routine. In relation to exercising, those who exercise presented lower symptoms of depression and anxiety than those who did not exercise. For time spent getting information about COVID-19 pairwise comparisons were performed using Fisher’s LSD test. The results indicated that those who spent an hour or less with this purpose presented lower sum scores in GAD-7 and PHQ-9 than those who avoid to look information about the topic (GAD-7 sum scores mean difference = 2.45, *p* < .001; PHQ-9 sum scores mean difference = 2.82, *p* < .001) and those who spent more than an hour getting information about COVID-19 (t GAD-7 sum scores mean difference = 2.11, *p* < .001; total PHQ-9 scores mean difference = 1.89, *p* < .001). No significant mean differences in total were found for those who avoid getting information and those who spent more than an hour getting information (GAD-7 sum scores mean difference = 0.34, *p* = .49; PHQ-9 sum scores mean difference = 0.92, *p* = .07).

**Table 2:** Behaviors during confinement and group differences of GAD-7 and PHQ-9 total sum scores.

Variable			GAD7			PHQ9		
		*n* (%)	Mean (SD)	Test	*p*	Mean (SD)	Test	*p*
Daily routine				6.09	< .001		7.17	< .001
	No	266(35)	7.79(5.61)			7.80(5.94)		
	Yes	489(64.4	5.33(4.69)			4.78(4.67)		
Exercise				-4.76	< .001		-7.51	< .001
	No	283(37.3)	6.85(5.39)			6.88(5.51)		
	Yes	476(62.7)	5.11(4.53)			4.11(4.53)		
Social Contact				1.63	.19		2.72	.06
	Less than 3 persons	155(15.2)	6.57(5.37)			6.85(6.31)		
	From 4–8 persons	353(46.5)	6.35(5.20)			5.69(5.23)		
	More than 9 persons	270 (35.6)	5.72(4.82)			5.53(4.78)		
Time spent focusing on information about the COVID-19				18.39	< .001		18.55	< .001
	Avoid the topic	164(21.6)	7.34(5.74)			7.36(6.58)		
	One hour or less	314 (41.4)	4.89(4.60)			4.54(4.47)		
	More than an hour	281(37)	7.00 (5.07)			6.43(5.10)		

^a^ *t-*statistic for variables with two levels and *F-* statistic for variables with more than two levels

### Behavioral and sociodemographic variables associated with depression and anxiety

Those variables presenting significant differences in the previous analyses were included in a two-step hierarchical regression analysis as predictors of GAD-7 sum scores and PHQ-9 sum scores separately. Sociodemographic variables were first included in a model, and behavioral ones were subsequently included to observe their contribution to the final model. Region was considered as a dichotomous variable, based on the previous result indicating that persons from Coastal have greater significant scores that the other two regions.

In relation to anxiety, in the first step of the regression model gender and region were included as predictors, and the regression model was statistically significant, *F* (2, 753) = 15.33, *p* < .001. The model explained 3.9% of the variance. Both variables significantly contributed to the model. In the second step, in which behaviors during the confinement were included in the model (maintaining regular schedule, exercising and time spent getting information about the COVID-19) along with sociodemographic variables, the model was statistically significant, *F* (6, 749) = 16.58, *p* < .001, and significantly improved explaining 11.8% of the variance of the GAD-7 total sum scores. The resulting unstandardized coefficients for the hierarchical linear regression analysis for variables predicting GAD-7 total sum scores can be found in Table 3.

**Table 3:** Hierarchical linear regression analysis (unstandardized regression coefficients) for variables predicting GAD-7 total sum scores.

Predictor	*b*	*p*	*B*	*sr*^*2*^	*sr*^*2*^	Fit	Difference
			95% CI [LL, UL]		95% CI [LL, UL]		
**Model 1**						*R*^*2*^ = .039** 95% CI [.02,.07]	
(Intercept)	7.41	< .001	[6.84, 7.98]				
Female vs. Male	-1.80	< .001	[-2.52, -1.07]	.03	[.01, .05]		
Coastal vs. Other regions	-0.87	0.017	[-1.61, -0.13]	.01	[-.00, .02]		
**Model 2**						*R*^*2*^ = .118**	Δ*R*^*2*^ = .078**
						95% CI [.07,.16]	95% CI [.04, .11]
(Intercept)	7.60	< .001	[6.72, 8.48]				
Female vs. Male	-1.29	< .001	[-2.01, -0.58]	.01	[-.00, .03]		
Coastal vs. Other regions	-0.68	.054	[-1.40, 0.04]	.00	[-.00, .01]		
Not regular vs. Regular schedule	-1.89	< .001	[-2.64, -1.15]	.03	[.01, .05]		
Not exercising vs. exercising	-0.84	.033	[-1.59, -0.08]	.01	[-.00, .02]		
One hour or less vs. More than an hour	1.75	< .001	[0.95, 2.54]	.02	[.00, .04]		
One hour or less vs. Avoid the topic	1.79	< .001	[0.84, 2.74]	.02	[-.00, .03]		

*Note*. A significant *b*-weight indicates the semi-partial correlation is also significant. *b* represents unstandardized regression weights. *sr*^*2*^ represents the semi-partial correlation squared. *LL* and *UL* indicate the lower and upper limits of a confidence interval, respectively.

** indicates p < .01.

In relation to depression, in the first step, the model was statistically significant, *F* (2, 753) = 21.62, *p* < .001, age and region significantly contributed to explain depressive symptoms, with the model explaining 5.4% of the variance. In the second step in which variables presenting behaviors during the confinement were added, the model was statistically significant, *F* (6, 749) = 24.73, *p* < .001, and accounted for 17% of the variance of the PHQ-9 total sum scores. All the variables significantly contributed to the model. The resulting unstandardized coefficients for the hierarchical linear regression analysis for variables predicting PHQ-9 total sum scores can be found in Table 4.

**Table 4:** Hierarchical linear regression analysis (unstandardized regression coefficients) for variables predicting PHQ-9 total sum scores.

Predictor	*b*	*p*	*b*	*sr*^*2*^	*sr*^*2*^	Fit	Difference
			95% CI		95% CI		
			[LL, UL]		[LL, UL]		
**Model 1**						*R*^*2*^ = .054**	
						95% CI [.03,.09]	
(Intercept)	7.36	< .001	[6.78, 7.95]				
Female vs. Male	-1.96	< .001	[-2.70, -1.21]	.03	[.01, .06]		
Coastal vs. Other regions	-1.43	< .001	[-2.19, -0.67]	.02	[-.00, .04]		
**Model 2**						*R*^*2*^ = .166**	Δ*R*^*2*^ = .111**
						95% CI	95% CI
						[.11,.21]	[.07, .15]
(Intercept)	8.16	< .001	[7.27, 9.04]				
Female vs. Male	-1.24	< .001	[-1.96, -0.52]	.01	[-.00, .03]		
Coastal vs. Other regions	-1.10	< .001	[-1.82, -0.37]	.01	[-.00, .02]		
Not regular vs. Regular schedule	-2.34	< .001	[-3.09, -1.59]	.04	[.02, .07]		
Not exercising vs. exercising	-1.76	< .001	[-2.53, -1.00]	.02	[.00, .04]		
One hour or less vs. More than an hour	1.35	< .001	[0.55, 2.15]	.01	[-.00, .03]		
One hour or less vs. Avoid the topic	1.87	< .001	[0.91, 2.82]	.02	[-.00, .03]		

*Note*. A significant *b*-weight indicates the semi-partial correlation is also significant. *b* represents unstandardized regression weights. *sr*^*2*^ represents the semi-partial correlation squared. *LL* and *UL* indicate the lower and upper limits of a confidence interval, respectively.

** indicates p < .01.

## Discussion

The current study aimed to characterize the mental health of an Ecuadorian population that was being surveilled epidemiologically by the health department as a result of either being diagnosed with COVID-19 or having shown symptoms consistent with the disease after a confirmed exposure, as well as to identify behaviors associated with their mental health. Results indicated that there is a very high prevalence of persons presenting at least mild symptoms of anxiety (58.1%) and depression (52.6%). Females and patients from Coastal regions presented with higher levels of depression and anxiety. Maintaining a daily routine and spending one hour or less daily seeking information were associated with lower anxiety and depression, while exercising was associated with lower levels of depression and anxiety. These results about prevalence of anxiety/depression are higher than those presented by Zhang et al. [12] with a similar population, participants which had a COVID-19 diagnosis or were under mandatory quarantine in China. In that study, 37.4% of the participants endorsed at least mild anxiety and up, while 47.6%, endorsed symptoms of at least mild depression and up. However, when considering the percentage of those endorsing moderate to severe symptoms the percentage presented in Zhang’s et al. [12] study was slightly higher than the presented in our study (21.5% vs. 20.3%). The percentage of participants endorsing moderate to severe anxiety symptoms was higher in our study than in Zhang’s et al. [12] study (22.5% vs 14%). In spite of cultural differences, the number of people presenting emotionally distress during the pandemic for COVID-19 appears to be grossly consistent. This information might prove useful for other countries planning a mental health response to current circumstances, especially taking in consideration the possible long-term mental health effects like chronic depression and PTSD, as it was observed in survivors of the Middle East respiratory syndrome (MERS) in South Korea [31].

The results of our investigation show that the prevalence found among COVID-19 patients is higher than the reported previously in Ecuador [32]. The Prevalence of major depressive disorders was 6.2% while generalized anxiety and panic disorders ranged from 0.2 to 2.2%. [32]. On the other hand, a recently published study suggest that younger populations are at greater risk of developing depression and anxiety after experiencing a shocking and stressing event as reported in 2020 [33].

The present study also revealed that some sociodemographic variables are associated with higher levels of depression and/or anxiety in patients diagnosed or suspected to have COVID-19. Being woman was associated with both, greater levels of depression and anxiety, and the difference in mean scores approximated two points in both conditions. For reference, a two-point increase in PHQ-9 or GAD-7 score could move a person from the mild to the moderate range, or from the latter to the severe range. This result is consistent with a study by Liu et al. [34] that found women reported a higher number of cognitive and mood symptoms in a hardest-hit area of China. Another factor associated with levels of depression and anxiety was the area of residence. Participants from Coastal region were more affected emotionally, and this makes sense given the progression of the disease in the country. Coastal areas, particularly Guayaquil, presented a higher number of cases and higher mortality rates in comparison to the other regions during April 2020 as result of the COVID-19 pandemic [23].

This study also found several behaviors associated with better mental health for those diagnosed or suspected to have COVID-19: exercising, maintaining daily routines, and time spent focusing on information about the COVID-19. The relationship between exercise and mental health is well-established in the literature [35, 36] and it is unsurprising to find this association in the current data, though reassuring because it is a modifiable protective factor that can be promoted and easily instantiated during the pandemic. Conversely, a lack of physical activity has been associated with the development of psychological disorders, which underscores the importance of promoting exercise during the pandemic [37]. Likewise, daily routine development and maintenance are a staple of many psychotherapeutic interventions for anxiety and depression, and the negative feelings stemming from isolation and the breakdown of normal routine in quarantine conditions has been documented extensively in the literature [6, 37]. Lastly, the current study found an association between information seeking about COVID-19 and mental health. This is consistent with results from Nguyen et al. [38] who found health literacy was associated with lower levels of depression and higher health-related quality of life in patients who had suspected COVID-19 symptoms. Of note, when analyzing the direct relationship between information seeking and mental health in this study, results showed that maintained informed about the COVID-19 is beneficial, but anything over an average of an hour a day might produce distress. Other studies, have indicated similar associations, in Germany the limit was two hours and a half [15] while in China [9] it was more than three hours. The difference in results might respond to the differences in social and cultural contexts in which studies were conducted and the evolution of the pandemic, however all the results support the fact that the excess of time getting information about the COVID-19 is related with the presence of psychological distress. These results support what has been recommended by the WHO [19] for maintaining mental health during the pandemic, which mentioned that it is essential to minimize the search for information of COVID-19 and focus on information on prevention and self-care practices, additionally, searching from rigorous and authoritative sources and no more than twice a day. Of note, our results indicate that the number of people with whom remote contact is maintained is not associated with psychological distress. Probably, the quality of the contact is a more relevant factor.

Regression analysis demonstrated that modifiable behaviors explained a significant amount of variance of PHQ-9 and GAD-7 sum scores when they are controlled by gender and region. That denotes the relevance of those behaviors as possible focus of attention for interventions for people in confinement. Such interventions might be taking care to promote exercising, maintaining daily routines, and inform about COVID-19, but restrict the time focusing on information to an hour or less in surveilled population.

This study has limitations. Due to the cross-sectional nature of this study it is no possible to establish causal conclusions, for example, whether symptoms of depression and/or anxiety will remit when the person no longer presents physical signs related with COVID-19 or if previous levels of anxiety and depression in women explain the scores obtained by this group. Furthermore, participants were not asked about premorbid psychiatric disorders and/or personality characteristics that may act as risk factors for the development of anxiety and/or depression in the face of new stressors. Lacking an ability to describe one´s emotional experience and maintain adequate self-regulation, as is the case in alexithymia and sensory processing difficulties, have been found to be associated with depression [39–42]. Future studies should obtain more information about the existing psychological characteristics of people diagnosed and/or suspected to have COVID-19 in order to ascertain possible relationships with the onset of anxiety/depression. In addition, taking into account that the study was carried out at the beginning of the pandemic and at that time the number of confirmed infected and deaths was still low, the present results represent only a snapshot of the situation of the persons under epidemiological surveillance at the time when the study was conducted. It is likely that the situation changed as the number of infected and confirmed deaths increased. Finally, responses were collected only from those patients who were willing to read the online survey or who were healthy enough to understand and to be able to respond to the online survey tool during follow-up.

## Conclusions

This study identified behaviors and sociodemographic traits associated with anxiety and depression in a group of participants with either a diagnosis or symptoms of COVID-19 in Ecuador. Being female and living in the Coastal region were associated with higher levels of symptoms of anxiety/depression, but more importantly behaviors like maintaining a routine, information seeking, and exercise were protective factors for poor mental health. This information can help shape public health policy not only by noting which populations are more vulnerable to be negatively affected by COVID-19, but also by suggesting possible strategies to mitigate the psychological effects. Behaviors during a pandemic are modifiable factors susceptible to public health interventions. It is evident that there is need for the implementation of programs to reduce psychological distress in the population under epidemiological surveillance. As suggested by Brooks et al. [6], this interventions should guarantee basic needs fulfilment, provision of adequate information about the disease and treatment, offering professional mental health support, maintaining communication with the own social network, and promotion of activities to reduce the boredom produced by staying in quarantine.

## Supporting information

S1 Dataset Anonymized data set. Minimal information to replicate the study findings.
(XLSX)
